# Can AI Mitigate Bias in Writing Letters of Recommendation?

**DOI:** 10.2196/51494

**Published:** 2023-08-23

**Authors:** Tiffany I Leung, Ankita Sagar, Swati Shroff, Tracey L Henry

**Affiliations:** 1 Department of Internal Medicine (adjunct) Southern Illinois University School of Medicine Toronto, ON Canada; 2 JMIR Publications Toronto, ON Canada; 3 CommonSpirit Health Chicago, IL United States; 4 Creighton University School of Medicine Omaha, NE United States; 5 Division of Internal Medicine Thomas Jefferson University Philadelphia, PA United States; 6 Department of Medicine Emory University School of Medicine Atlanta, GA United States

**Keywords:** sponsorship, implicit bias, gender bias, bias, letters of recommendation, artificial intelligence, large language models, medical education, career advancement, tenure and promotion, promotion, leadership

## Abstract

Letters of recommendation play a significant role in higher education and career progression, particularly for women and underrepresented groups in medicine and science. Already, there is evidence to suggest that written letters of recommendation contain language that expresses implicit biases, or unconscious biases, and that these biases occur for all recommenders regardless of the recommender’s sex. Given that all individuals have implicit biases that may influence language use, there may be opportunities to apply contemporary technologies, such as large language models or other forms of generative artificial intelligence (AI), to augment and potentially reduce implicit biases in the written language of letters of recommendation. In this editorial, we provide a brief overview of existing literature on the manifestations of implicit bias in letters of recommendation, with a focus on academia and medical education. We then highlight potential opportunities and drawbacks of applying this emerging technology in augmenting the focused, professional task of writing letters of recommendation. We also offer best practices for integrating their use into the routine writing of letters of recommendation and conclude with our outlook for the future of generative AI applications in supporting this task.

## Introduction

Letters of recommendation play a significant role in higher education and career progression, particularly for women and underrepresented groups in medicine and science. Letters of recommendation include any letter written to support or sponsor an individual for a job [1,2], internship [3], or training position [4]; a scholarship or grant; an award or recognition; a promotion; or other important professional milestones. For example, letters of support for a job application may be used in so-called *round 1* selection stages, even before a candidate interviews for a position. This means that such letters and evaluations, as well as the language used to describe a candidate, can significantly, even if unintentionally, influence a hiring committee's consideration of an individual’s candidacy. Already, there is evidence to suggest that written letters of recommendation contain language that expresses implicit biases, or unconscious biases [5,6], and that these biases occur for all recommenders regardless of the recommender’s sex [7]. Given that all individuals have implicit biases that may influence language use, there may be opportunities to apply contemporary technologies, such as large language models (LLMs) or other forms of generative artificial intelligence (AI), to augment and potentially reduce implicit biases in the written language of letters of recommendation. Although AI has been used to analyze recommendation letter content for bias via, for example, natural language processing and sentiment analysis [8] or automated text mining [9,10], there remains an unexplored potential opportunity to apply AI to generate letters, especially with the aim of reducing bias.

As of May 2023, some of the authors had one-on-one conversations with medical faculty peers or leaders and even heard conference plenary speakers explicitly endorse subscribing to generative AI services, such as ChatGPT Plus [11], to help them specifically with writing letters of recommendation. It is very likely that there are many professionals who apply such services, yet little to no exploration of the potential opportunities and pitfalls has been reported on this application of generative AI. In this editorial, we provide a brief overview of existing literature on the manifestations of implicit bias in letters of recommendation, with a focus on academia and medical education. We then highlight potential opportunities and drawbacks of applying this emerging technology in augmenting the focused, professional task of writing letters of recommendation. We also offer best practices for integrating their use into the routine writing of letters of recommendation and conclude with our outlook for the future of generative AI applications in supporting this task. For the purposes of this editorial, we focus on letters of recommendation, although the presence of bias in performance evaluations and assessments [12-15], especially in medical training, is also a well-recognized phenomenon. It may be possible to apply some of the key points raised in this editorial similarly to writing performance evaluations.

## Implicit Bias in Letters of Recommendation

Implicit bias is a type of bias that arises from unconscious associations and stereotypes about members of a social group. Often, bias is based on gender, race, ethnicity, ability, language proficiency, or any aspect of one’s identity. Gendered language usage occurs in medicine, health care, and professions and areas beyond our usual areas as physicians; the World Bank noted in a 2019 report that “[a]ttitudes toward women are also influenced by gendered languages…gendered languages could translate into outcomes like lower female labor force participation” [16].

Gendered terms are words that are associated with a specific gender. Various studies have noted that gendered language appears in letters of recommendation for academic faculty, science, and medicine [5]. Specifically, categories of terms include communal terms (eg, “caring,” “nurturing,” “attentive,” or “kind”), which occur more frequently in recommendation letters for women, and agentic terms (eg, “confident,” “assertive,” “outspoken,” or “ambitious”), which occur more frequently in recommendation letters for men [5]. In a study by Trix and Psenka [6], the adjective “successful” occurred in 7% and 3% of letters for men and women, respectively, while the nouns “accomplishment” and “achievement” occurred in 13% and 3% of letters for men and women, respectively. For women applicants, “compassionate” and “relates well to patients and staff at all levels” stood out (16% vs 4% in letters for women and men, respectively) [6].

Less recognized categories of descriptors include hedging language, doubt-raisers, and grindstone language [6]. Such language is more often applied to women in recommendation letters than to men. Doubt-raising language includes negative, potentially negative, hedging, unexplained, or irrelevant comments and faint praise [6,7]. Examples of doubt-raising language include “while she has not done”; “while not the best student I have had”; and “bright, enthusiastic, he responds well to a minimum amount of supervision.” Examples of hedging include “it appears that” or “now that she has chosen,” and an example of faint praise is “she worked hard on projects that she enjoys.” Grindstone language implies that an individual is hardworking because of a need to compensate for a shortcoming in their ability (eg, “hardworking,” “conscientious,” or “dedicated”) [17]. For example, “She is a superb experimentalist – very well organized, thorough and careful in her approach to research” [6].

## Tools to Identify Implicit Bias in Language

Out-of-the-box tools to help with identifying commonly used categories of words are readily available for research purposes. One commonly used tool in text analysis is Linguistic Inquiry and Word Count (LIWC) [18,19]. LIWC offers text analysis tools based upon established LIWC dictionary categories [20] that can be augmented with user-defined dictionaries; Madera et al [5] validated added dictionaries of communal and agentic terms in their study of gendered language in recommendation letters [21]. Additional researchers have also created, although not yet validated, 5 additional user-defined dictionaries, including grindstone traits, ability traits, standout adjectives, research terms, and teaching terms [1,6,21-23]. LIWC usage typically requires a paid license for users, and LIWC offers its dictionaries in more than 15 languages.

Additional text analysis and processing techniques also can be applied in various ways to recommendation letters to identify biased language. Such approaches can involve using pre-established dictionaries of terms (eg, from LIWC), performing text mining [9] or topic modeling [24], or applying natural language processing packages [8].

Real-time integrated tools to identify biased language are available in productivity platforms. For example, the #BiasCorrect plug-in in Slack works “like spell check but for gender bias, this plug-in will flag your unconscious bias to you in real-time and offer up bias-free alternatives for you to consider instead” [25]. Integrated tools, extensions, or plug-ins are appealing; however, no such real-time tool exists yet in a text processing program. There are also several websites where users can copy and paste individual words or short chunks of text into a web-based form to identify which words are used more often for women or men and, perhaps, even in certain disciplines [26,27]. However, these are stand-alone tools that may serve as more of a curiosity rather than a routinely usable support in the recommendation letter writing workflow. Additionally, all of these existing tools share the same feature of first depending on the human generation of language and then reactively providing feedback if the writer is aware of the tool and uses it with a specific intention.

## LLMs for Letters of Recommendation

### Overview of LLMs

The concept of AI augmentation of human tasks is not new; augmentation “is where employers create workplaces that combine smart machines with humans in close partnerships—symbiotically taking advantage of both human intelligence and machine intelligence. In other words, the AI system is used to complement the capabilities of a human worker (or vice versa)” [28]. Similarly, AI augmentation of writing letters of recommendation can offer a pathway to improve letter writing while keeping the human in the loop. Briefly, LLMs are based on a transformer model, a neural network architecture that initially involves a pretraining stage of self-supervised learning from a large amount of unannotated data. Subsequently, in a fine-tuning stage, further training on a smaller, task-specific data set can be done to facilitate specific tasks [29]. Since the initial general popularity of LLMs during late 2022, with OpenAI’s ChatGPT [30], countless additional LLMs have been developed and launched. Notably, there are also free, open-source models available for research or commercial use, like Meta’s Llama 2 [31].

### Training an LLM

Any algorithm or AI is only as good as the training data with which the model is trained. LLMs have already been shown to, for example, generate statements that have certain political leanings [32,33] or have cultural biases [34,35]. If the training data are biased, because of the probabilistic nature of the language generated in an LLM, that bias can be perpetuated or amplified in prompted outputs. Nevertheless, the potential of LLMs to support the task of recommendation letter writing is still a major opportunity that cannot be ignored.

Using open-source LLMs to train one's own generative AI on a set of one’s own recommendation letters is a possibility, but this perhaps is limited by the size of the training set and the potential of unintentionally amplifying one's own implicit biases. During a workshop at the American Medical Informatics Association’s Annual Symposium in 2020, on the topic of bias in recommendation letters, one advanced career academic faculty member with 3 decades of experience in their field reflected on their writing of over 200 recommendation letters [36]. At that time, a named entity recognition approach to identifying key words offered a preliminary glimpse at one individual’s writing patterns.

### Increasing Efficiency

Improving the efficiency of recommendation letter writing can be especially valuable in easing the burden of this task for the small proportion of underrepresented groups who are in top leadership positions in medicine and scientific fields. For example, in medicine, although the proportion of women department chairs has increased over the last decade, still only 18% are women; the proportion of women medical school deans has barely shifted since 2012, increasing from 16% to 18% in 2018 [37]. In academia, when promotion from associate professor to full professor requires letters of recommendation from individuals with a rank identical to that being sought, this burden can be especially amplified for women faculty among the highest academic ranks. Fortunately, the gender gap at the full-time professor level has narrowed over the past decade, yet still only 25% of full professors are women as of 2018 [38,39].

Although no biased language checker plug-ins are available in word processing software, some LLMs have the capability to potentially ingest one or more files in various formats. Conceivably, a curriculum vitae in PDF format could be provided as part of a prompt. Afterward, with thoughtful prompts, the LLM could generate relevant portions of a recommendation letter for a writer to use. Putting the energy of generation on the AI, with the human in a position of writing, could be a time-saver. Alternatively, a human writing a rough draft can also prompt AI to refine and polish the language of the recommendation letter. There are more ways that AI can augment the recommendation letter writing process, and in all cases, these would help with the efficiency of generating the letters for busy faculty or those who may need extra support to write professionally and clearly in the language required for the letter. Moreover, as efficiency improves, a diverse range of letter writers can be created across the gender spectrum, thus alleviating burdens and fostering a culture of thoughtful language that emphasizes the merits and potential of candidates for promotion or leadership.

### Cautionary Notes

Some additional notes of caution are warranted for anyone considering using generative AI to help them with writing recommendation letters. In scientific publishing, there is almost no remaining controversy as to whether generative AI can coauthor a manuscript (it should not [40-42]). The arguments for no generative AI coauthorship center on accountability. The sense of accountability for the factual content of a written document is self-evident. Publishers either ban generative AI use by authors in generating portions of a manuscript or permit it to a limited extent and with required disclosure and transparency. No analogous guidelines exist for writing recommendation letters, especially since it is a common practice that recommendation letter writers can recycle their letters as templates for another similar letter, or some letter writers ask the candidate to draft a first version of the letter. Although we do not expect letter writers to disclose generative AI use, accountability for the outputs used in an official final recommendation letter lies solely with the signer of the letter.

Additionally, the focus here has been on recommendation letter writing. The other half of this process is recommendation letter reading and interpretation. Regardless of self-generated text or AI-assisted generation of text, there is a history of bias in AI-supported hiring [43]. Even human screeners are not immune to this bias, tending to carry biases when they, for example, perceive a name to be identifying a person's gender or race [44,45]. This half of the issue on recommendation letter interpretation and, more generally, on AI-supported hiring processes has been the focus of recent regulation in New York City [46].

Finally, we cannot emphasize enough that the aim is to reduce bias in language, not to reduce how often women candidates are written about as being “caring” or “nurturing.” In medicine, all physician candidates would ideally embody these traits, among others, in comparable ways that are needed for them to be successful in the target roles they are being recommended for.

## Conclusion

Overall, we are optimistic about the potential of generative AI in augmenting recommendation letter writing. Naturally, the opportunities we raise in this editorial are not without their potential limitations. One major counterargument is that the application of any technology to this specific task does not (or cannot) address the underlying problems that racism, stereotyping, and various forms of bias and discrimination are deeply rooted in systemic and organization structure. As a result, the potential for gender bias in AI remains possible [47]. We agree with this position and see the application of technology, in the ways described in this editorial, as a supplementary tool or option for existing programs and initiatives around implicit bias recognition and management [48], rather than as a replacement or substitution. Additionally, although this editorial does not address other professional documents that may benefit from technological augmentation, there is evidence to suggest that biased language appears in evaluations of trainees [49], including subjective evaluations for students applying to residency programs [24]; qualitative evaluations of residents and students [12,50]; student, resident, and fellow evaluations of faculty physicians [9]; and more [51,52]. Racial bias in evaluations also is problematic [53-55].

In a future investigation, we aim to further determine what practices current faculty and physicians are using in the AI augmentation of their writing of letters of recommendation. There may also be opportunities to computationally determine prompts that best facilitate recommendation letter writing with minimal implicit bias [56] or to fine-tune an LLM based on a large corpus of recommendation letters. We look forward to the advancements that medical and scientific education and career advancement processes can benefit from, including new technological tools, like generative AI, to overcome systemic biases for women and underrepresented groups in their respective disciplines. AI augmentation can be a tool when utilized mindfully and with caution, improving one letter of recommendation at a time. This has the potential to address and mitigate systemic biases, especially when equity in medical and scientific careers is at stake [57,58].

## Abbreviations

AI: artificial intelligence
LIWC: Linguistic Inquiry and Word Count
LLM: large language model

## References

[1] Schmader T, Whitehead J, Wysocki VH (2007). A linguistic comparison of letters of recommendation for male and female chemistry and biochemistry job applicants. Sex Roles.

[2] Bernstein RH, Macy MW, Williams WM, Cameron CJ, Williams-Ceci SC, Ceci SJ (2022). Assessing gender bias in particle physics and social science recommendations for academic jobs. Soc Sci.

[3] Houser C, Lemmons K (2017). Implicit bias in letters of recommendation for an undergraduate research internship. J Furth High Educ.

[4] Grimm LJ, Redmond RA, Campbell JC, Rosette AS (2020). Gender and racial bias in radiology residency letters of recommendation. J Am Coll Radiol.

[5] Madera JM, Hebl MR, Martin RC (2009). Gender and letters of recommendation for academia: agentic and communal differences. J Appl Psychol.

[6] Trix F, Psenka C (2003). Exploring the color of glass: Letters of recommendation for female and male medical faculty. Discourse & Society.

[7] Madera JM, Hebl MR, Dial H, Martin R, Valian V (2018). Raising doubt in letters of recommendation for academia: Gender differences and their impact. J Bus Psychol.

[8] Sarraf D, Vasiliu V, Imberman B, Lindeman B (2021). Use of artificial intelligence for gender bias analysis in letters of recommendation for general surgery residency candidates. Am J Surg.

[9] Heath JK, Weissman GE, Clancy CB, Shou H, Farrar JT, Dine CJ (2019). Assessment of gender-based linguistic differences in physician trainee evaluations of medical faculty using automated text mining. JAMA Netw Open.

[10] Alexander CS (2022). Text mining for bias: A recommendation letter experiment. American Business Law Journal.

[11] Introducing ChatGPT Plus. OpenAI.

[12] Klein R, Julian KA, Snyder ED, Koch J, Ufere NN, Volerman A, Vandenberg AE, Schaeffer S, Palamara K, Gender Equity in Medicine (GEM) workgroup (2019). Gender bias in resident assessment in graduate medical education: Review of the literature. J Gen Intern Med.

[13] Arora VM, Carter K, Babcock C (2022). Bias in assessment needs urgent attention-no rest for the "Wicked". JAMA Netw Open.

[14] Mamtani M, Shofer F, Scott K, Kaminstein D, Eriksen W, Takacs M, Hall AK, Weiss A, Walter LA, Gallahue F, Yarris L, Abbuhl SB, Aysola J (2022). Gender differences in emergency medicine attending physician comments to residents: A qualitative analysis. JAMA Netw Open.

[15] Dayal A, O'Connor DM, Qadri U, Arora VM (2017). Comparison of male vs female resident milestone evaluations by faculty during emergency medicine residency training. JAMA Intern Med.

[16] (2019). Gendered languages may play a role in limiting women’s opportunities, new research finds. The World Bank.

[17] Valian V (1999). Why So Slow?: The Advancement of Women.

[18] Pennebaker JW, Booth RJ, Boyd RL, Francis ME (2015). Linguistic Inquiry and Word Count: LIWC2015. LIWC.

[19] Hovy D (2021). Text Analysis in Python for Social Scientists: Discovery and Exploration.

[20] Welcome to LIWC-22. LIWC.

[21] Miller DT, McCarthy DM, Fant AL, Li-Sauerwine S, Ali A, Kontrick AV (2019). The standardized letter of evaluation narrative: Differences in language use by gender. West J Emerg Med.

[22] Dutt K, Pfaff DL, Bernstein AF, Dillard JS, Block CJ (2016). Gender differences in recommendation letters for postdoctoral fellowships in geoscience. Nat Geosci.

[23] Friedman R, Fang CH, Hasbun J, Han H, Mady LJ, Eloy JA, Kalyoussef E (2017). Use of standardized letters of recommendation for otolaryngology head and neck surgery residency and the impact of gender. Laryngoscope.

[24] Turrentine FE, Dreisbach CN, St Ivany AR, Hanks JB, Schroen AT (2019). Influence of gender on surgical residency applicants' recommendation letters. J Am Coll Surg.

[25] #BiasCorrect install. Catalyst.

[26] Schmidt B Gendered language in teaching evaluations. Ben Schmidt blog.

[27] Forth T Gender bias calculator. Tom Forth blog.

[28] Miller SM, Davenport T (2022). AI and the future of work: What we know today. Tom Davenport.

[29] Shen Y, Heacock L, Elias J, Hentel KD, Reig B, Shih G, Moy L (2023). ChatGPT and other large language models are double-edged swords. Radiology.

[30] Introducing ChatGPT. OpenAI.

[31] (2023). Meta and Microsoft introduce the next generation of Llama. Meta AI.

[32] Rozado D (2023). The political biases of ChatGPT. Soc Sci.

[33] Hartmann J, Schwenzow J, Witte M The political ideology of conversational AI: Converging evidence on ChatGPT's pro-environmental, left-libertarian orientation. arXiv..

[34] Cao Y, Zhou L, Lee S, Cabello L, Chen M, Hershcovich D Assessing cross-cultural alignment between ChatGPT and human societies: An empirical study. arXiv..

[35] Ferrara E Should ChatGPT be biased? Challenges and risks of bias in large language models. arXiv..

[36] Leung TI, Ancker JS, Cimino JJ, Ross H, Wu H (2020). S104: panel - an unseen art: Writing letters of support and nomination to promote diversity, equity, and inclusion in informatics.

[37] The state of women in academic medicine. Association of American Medical Colleges.

[38] Joseph MM, Ahasic AM, Clark J, Templeton K (2021). State of women in medicine: History, challenges, and the benefits of a diverse workforce. Pediatrics.

[39] Richter KP, Clark L, Wick JA, Cruvinel E, Durham D, Shaw P, Shih GH, Befort CA, Simari RD (2020). Women physicians and promotion in academic medicine. N Engl J Med.

[40] Jackson J, Landis G, Baskin PK, Hadsell KA, English M, CSE Editorial Policy Committee (2023). CSE guidance on machine learning and artificial intelligence tools. Science Editor.

[41] Zielinski C, Winker MA, Aggarwal R, Ferris LE, Heinemann M, Lapeña JF Jr, Pai SA, Ing E, Citrome L, Alam M, Voight M, Habibzadeh F, WAME Board (2023). Chatbots, generative AI, and scholarly manuscripts. World Association of Medical Editors.

[42] Stokel-Walker C (2023). ChatGPT listed as author on research papers: many scientists disapprove. Nature.

[43] Drage E, Mackereth K (2022). Does AI debias recruitment? Race, gender, and AI's "Eradication of Difference". Philos Technol.

[44] Steinpreis RE, Anders KA, Ritzke D (1999). The impact of gender on the review of the curricula vitae of job applicants and tenure candidates: A national empirical study. Sex Roles.

[45] Wenneras C, Wold A (1997). Nepotism and sexism in peer-review. Nature.

[46] Automated employment decision tools. NYC311.

[47] Thakur V Unveiling gender bias in terms of profession across LLMs: Analyzing and addressing sociological implications. arXiv..

[48] Rodriguez N, Kintzer E, List J, Lypson M, Grochowalski JH, Marantz PR, Gonzalez CM (2021). Implicit bias recognition and management: Tailored instruction for faculty. J Natl Med Assoc.

[49] Hemmer PA, Karani R (2019). Let's face it: We are biased, and it should not be that way. J Gen Intern Med.

[50] Gerull KM, Loe M, Seiler K, McAllister J, Salles A (2019). Assessing gender bias in qualitative evaluations of surgical residents. Am J Surg.

[51] Smith DG, Rosenstein JE, Nikolov MC, Chaney DA (2018). The power of language: Gender, status, and agency in performance evaluations. Sex Roles.

[52] Sheffield V, Hartley S, Stansfield RB, Mack M, Blackburn S, Vaughn VM, Heidemann L, Chang R, Lukela JR (2022). Gendered expectations: the impact of gender, evaluation language, and clinical setting on resident trainee assessment of faculty performance. J Gen Intern Med.

[53] Ross DA, Boatright D, Nunez-Smith M, Jordan A, Chekroud A, Moore EZ (2017). Differences in words used to describe racial and gender groups in medical student performance evaluations. PLoS One.

[54] Rojek AE, Khanna R, Yim JWL, Gardner R, Lisker S, Hauer KE, Lucey C, Sarkar U (2019). Differences in narrative language in evaluations of medical students by gender and under-represented minority status. J Gen Intern Med.

[55] Stack TJ, Berk GA, Ho TD, Zeatoun A, Kong KA, Chaskes MB, Thorp BD, Ebert CS Jr, DeMason CE, Senior BA, Kimple AJ (2023). Racial and ethnic bias in letters of recommendation and personal statements for application to otolaryngology residency. ORL J Otorhinolaryngol Relat Spec.

[56] Jiang Z, Xu FF, Araki J, Neubig G (2020). How can we know what language models know?. Trans Assoc Comput Linguist.

[57] Bates C, Gordon L, Travis E, Chatterjee A, Chaudron L, Fivush B, Gulati M, Jagsi R, Sharma P, Gillis M, Ganetzky R, Grover A, Lautenberger D, Moses A (2016). Striving for gender equity in academic medicine careers: A call to action. Acad Med.

[58] Leung TI, Barrett E, Lin TL, Moyer DV (2019). Advancing from perception to reality: How to accelerate and achieve gender equity now. Perspect Med Educ.

[59] Sagar A, Henry T, Shroff S, Leung TI Best practices: Reading between the lines to promote diversity, equity, and inclusion. SGIM Forum.

[60] Leung T, Sagar A, Henry TL, Shroff S (2023). SGIM2022: Recognizing and reducing bias in letters of support and performance evaluations in 360 degrees.

